# Editorial: Epstein-Barr Virus and multiple sclerosis

**DOI:** 10.3389/fimmu.2023.1330181

**Published:** 2024-01-05

**Authors:** Gunnar Houen, Klemens Ruprecht

**Affiliations:** ^1^ Department of Neurology, Rigshospitalet, Glostrup, Denmark; ^2^ Department of Neurology, Charité – Universitätsmedizin Berlin, corporate member of Freie Universität Berlin and Humboldt-Universität zu Berlin, Berlin, Germany

**Keywords:** Epstein - Barr Virus, multiple sclerosis, genetic factors, environmental factors, human herpes virus 6, gene-environment (GxE) interaction

Multiple sclerosis (MS) is an inflammatory demyelinating central nervous system (CNS) disease, which is thought to result from an interplay of genetic and environmental factors. MS has a female preponderance (approximately 3:1) and while genetic risk factors for MS include presence of the HLA-DRB1*1501 allele, non-infectious environmental risk factors comprise cigarette smoking, low vitamin D (vitD) levels and obesity in early life (1–4). Concerning infectious risk factors for MS, compelling evidence suggest that infection with the Epstein-Barr Virus (EBV) plays a causal role in the development of MS (5, 6); EBV may thus be regarded as a necessary, but not sufficient factor for the development of MS (2–5).

EBV, also called human herpes virus 4 (HHV4), is a B lymphotropic human gamma herpesvirus, infecting >90% of the world’s adult population (7, 8). Whereas primary EBV infection typically occurs during early childhood and is most often asymptomatic, if delayed until adolescence, primary EBV infection frequently manifests as infectious mononucleosis (IM) (7, 8).

Importantly, although MS can be considered as a rare complication of EBV infection, the precise mechanisms underlying the role of EBV in MS remain unknown (3, 4). Against this background, the articles collected in this Research Topic highlight several aspects of the association between EBV and MS.

Previous epidemiological findings indicating a tight association between IM and MS were further substantiated by a retrospective cohort study by Loosen et al. Leveraging data from a large German outpatient database, Loosen et al. found that the incidence of MS was approximately twice as high among persons who had experienced IM, with hazards ratios (HR) being largest in the age group 14-20 years and in males compared to females. As IM occurs during late primary EBV infection, this suggest that late primary EBV infection particularly increases the risk for MS.

Hedström comprehensively reviews current knowledge of genetic and environmental risk factors, and their interactions, in MS pathogenesis and places them in the context of EBV infection. Although evidence suggests that other established risk factors for MS act synergistically with EBV in the development of MS, the biological mechanisms underlying these interactions are only beginning to be resolved. Examples for possible mechanisms include that EBV uses HLA II as an entry receptor and evades host immunity depending on HLA types, smoking can promote EBV reactivation, vitD is important for control of EBV, and obesity weakens EBV control. Altogether, the review by Hedström highlights that, although challenging, a more comprehensive understanding of the interaction of various risk factors for MS may be an important approach towards a better understanding of the role of EBV in MS.

While the causative role of EBV in MS is well established, other viral infections may also play a (modifying) role. Lezhnyova et al. have analyzed the prevalence of antibodies to different human herpesviruses and the occurrence of genomic single nucleotide polymorphisms (SNPs) in MS patients and control persons. Whereas in patients with MS, antibodies to EBV had the highest seroprevalence among the investigated antiviral antibodies (CMV, HHV6, EBV and VZV), HHV6 Abs were found to be more frequent in patients with MS than in healthy controls. Regarding SNPs, statistically significant differences were found for *CD58*, *CD6* (patients vs controls), *CD40* (female vs male). Statistically significant differences in SNPs were also found in relation to HHV6 Ab positivity (*IL2RA, CD40*) and VZV Ab positivity (*STK11, CD40*), implying a possible role for these herpesviruses in MS, as has been reported earlier for HHV6A (9).

EBV infects epithelial cells using integrins as an entry receptor, but other membrane proteins and constituents may also play a role in relation to entry and exit. Furthermore, it has been hypothesized that EBV may infect CNS cells during disease processes. In this context, Rani et al. investigated basic aspects of the role of membrane cholesterol for astrocyte infection by EBV, showing a significant effect on EBV entry and gene expression in astroglial cells.

Finally, Hassani and Khan comprehensively review animal models of EBV infection, which may represent a pertinent approach to gain mechanistic insights into the role of EBV in MS. Humans are the only natural host for EBV, but humanized mice can be infected and recapitulate several aspects of IM and, when reconstituted with MS-associated HLA-DR types, might be used to study the influence of genetic risk factors for MS on parameters of EBV infection. Mice are naturally infected by murine herpes virus 68 (MHV68), which is a close relative of EBV and HHV8 (Kaposi Sarcoma Virus), and this can be used to study basic aspects of gamma herpesvirus biology, IM and some aspects of MS-like pathology. Furthermore, rabbits can be infected by EBV under some circumstances and infected B cells can enter the CNS, as is seen in MS. Primates like Rhesus monkeys and Japanese macaques are naturally infected by distant relatives of EBV, which recapitulate some aspects of MS-like disease.

Whereas the articles collected in this Research Topic contribute to paving the way towards a better understanding of the role of EBV in MS, many aspects of the association between EBV and MS currently remain unknown. MS is considered to be an autoimmune disease, but the frequency of autoantibodies in MS is low (4). Molecular mimicry has been described for several EBV proteins and human proteins, and epitope spreading and bystander activation/damage may also play important roles (3, 4, 10–12). CD20 antibodies, the most recent addition to the therapeutic management of MS, specifically target the major B cell populations harboring active or latent EBV, and other drugs may also target different stages of EBV infection (13). Altogether, further elucidation of the biological mechanisms underlying the role of EBV in MS highly likely will reveal important insights into the pathogenesis of MS. Given the strength of the evidence linking EBV and MS, strategies directed against EBV appear warranted in the treatment of patients with MS and, in our opinion, anti-EBV vaccine development should have a high priority.

## Author contributions

GH: Conceptualization, Supervision, Writing – original draft. KR: Conceptualization, Supervision, Writing – review & editing.
